# Bevacizumab therapy normalizes the pathological intraocular environment beyond neutralizing VEGF

**Published:** 2010-10-27

**Authors:** Rajesh K. Sharma, Anna T. Rogojina, K.V. Chalam

**Affiliations:** 1 Department of Ophthalmology, University of Florida, Jacksonville FL; 2 St. Jude Children’s Research Hospital, Memphis TN

## Abstract

**Purpose:**

Vascular endothelial growth factor (VEGF) plays a key role in neovascularization by stimulating the proliferation and migration of vascular endothelial cells. The anti-VEGF therapy bevacizumab acts by binding to VEGF and preventing its effects. However, this linear interaction represents only a partial view of the pathobiology of neovascular diseases and the anti-VEGF treatment. To obtain an integrated view of the processes involved in VEGF-related ocular pathologies, we applied a systems approach and investigated whether intravitreal bevacizumab injections have a global effect in normalizing the ocular physiology perturbed by the disease.

**Methods:**

We analyzed 90 analytes representing various pathophysiological processes in aqueous humor. The samples were obtained from eight patients receiving intravitreal bevacizumab injections for various ocular VEGF-related conditions. The samples were obtained before and after the injection and were analyzed using microbead technology developed by Luminex xMAP.

**Results:**

Forty-three analytes were detected above the sensitivity of the assay both in pre- and post-injection samples. Of these, normal values of 41 analytes were known and these analytes were further analyzed. The detected analytes included relevant markers such as VEGF, C reactive protein, glutathione, and cytokines. We identified 24 markers that were perturbed more than 1.5 fold in diseased samples (pre-injection) compared to normal levels. The levels of perturbed analytes were compared in post-treatment samples. The results demonstrated an unequivocal trend toward normalization in post-treatment samples.

**Conclusions:**

Our results show intraocular bevacizumab injections change the perturbed physiologic environment of the eye toward normalization. Its effects reached beyond neutralizing VEGF. The results also demonstrate that large-scale analysis of the aqueous, using a systems approach, could provide useful insight regarding ocular diseases, their pathophysiologies, and treatment responses.

## Introduction

Neovascularization, a common pathology in several diseases, results from complex interactions between pro and antiangiogenic cytokines involving multiple pathways. Of the various cytokine families that play roles in the development, maintenance, and remodeling of microcirculation, the vascular endothelial growth factor (VEGF) family is the most complex and most studied. Vascular endothelial growth factor stimulates the cell proliferation, migration, and survival of vascular endothelial cells [1,2]. Anti-VEGF therapy has become a widely accepted treatment for several diseases where neovascularization and permeability plays a pivotal role, including cancer and retinal disorders. Anti-VEGF therapy, including bevacizumab, acts by binding to VEGF and preventing its cellular effects. However, this linear interaction represents only a partial view of the pathobiology of the disease and treatment processes. Consequently, the classical concept of linear interactions is being replaced by the concept of networks of interactions, emphasizing the importance of interactions between different components of a biologic system [3]. With a large number of interacting components in the process of neovascularization, focus on a single or a small number of molecules imposes the risk making incomplete and flawed interpretations. For such a complex process, a systems biology approach can provide useful insights.

Beyond genetic and environmental determinants, diseases are characterized by a perturbed physiology. Therefore, methods providing deeper insights into physiologic states are essential in acquiring an integrated view of human disease [4]. Spectacular progresses in quantitative techniques, large-scale measurement methods, and integration between experimental and computational approaches has made it possible to understand organisms, their physiology, and pathobiology at the system level. Applications of such system-wide approaches to human biology, ushered in by the human genome project, are likely to open new opportunities in medicine.

We have previously demonstrated that using a microbead assay, a quantitative analysis of a large number of analytes in aqueous humor can provide useful system-wide information about the pathophysiological intraocular environment [5]. We can simultaneously monitor concurrent activities of multiple molecules using this approach, enabling a broader and unbiased view of biologic events. In the current study, we applied the same methodologies to investigate how VEGF-related diseases of the eye change the intraocular environment and whether anti-VEGF therapy has a system-wide effect on normalizing the perturbed ocular environment.

## Methods

### Samples

Aqueous humor was obtained from eight patients undergoing bevacizumab injections for various VEGF-related ocular pathologies. Of the eight patients, three were male and five were female. Two patients were treated for central retinal vein occlusion (CRVO), one for branch retinal vein occlusion (BRVO), two for clinically significant macular edema (CSME), and three for age-related macular degeneration (AMD). The samples were drawn sequentially on two different occasions from the same patient. The first sample (pre-treatment sample) was drawn just before the first intravitreal bevacizumab injection (0.125 mg), representing the pathological sample. The second sample (post-treatment sample) was drawn before the second intravitreal injection of bevacizumab, representing the effect of treatment on the aqueous profile after the eye was injected with 0.25 mg of bevacizumab. Aqueous humor (80–100 µl) was withdrawn through a limbal paracentesis site using a 27-gauge needle in a tuberculin syringe. Care was taken to avoid touching intraocular tissues and to prevent contamination of aqueous samples with blood. The samples were immediately frozen and stored at −80 °C. Patients with other ocular or systemic disease, such as inflammatory diseases, were excluded from the study.

Informed consent was obtained from the patients, and the research was in compliance with the tenets of the University of Florida and the Declaration of Helsinki for experiments involving human tissue.

### Multiplex analysis

Multiplex analysis was performed at Rules-Based Medicine (Austin, TX), which uses multi-analyte profiles (MAPs) based on powerful Luminex xMAP® (Luminex Corporation, Austin TX) technology to discover biomarker patterns within very small sample volumes. The aqueous samples were thawed at room temperature, vortexed, and spun at 13,000× g for 5 min to remove any precipitates. The maximum available volume (80–100 ul) was removed for MAP antigen analysis into a master microtiter plate. Using automated pipetting, an aliquot of each sample was introduced into one of the capture microsphere multiplexes of the human antigen MAP. These mixtures of sample and capture microspheres were thoroughly mixed and incubated at room temperature for 1 h. Multiplexed cocktails of biotinylated reporter antibodies for each multiplex were then added robotically and were thoroughly mixed. The mixture was then incubated for an additional 1 h at room temperature. Multiplexes were developed using an excess of streptavidin-phycoerythrin solution, which was thoroughly mixed into each multiplex and incubated for 1 h at room temperature. The volume of each multiplexed reaction was reduced by vacuum filtration and the volume increased by dilution into matrix buffer for analysis. Analysis was performed in a Luminex 100 instrument and the resulting data stream was interpreted using proprietary data analysis software (developed at Rules-Based Medicine and licensed to Qiagen Instruments, Valencia, CA). For each multiplex, both calibrators and controls were included on each microtiter plate. Eight-point calibrators were run in the first and last column of each plate and three-level controls were included in duplicate. Testing results were determined first for the high, medium, and low controls for each multiplex to ensure proper assay performance. Unknown values for each of the analytes localized in a specific multiplex were determined using four and five parameters and weighted and non-weighted curve fitting algorithms included in the data analysis package.

### Analysis

The value for each analyte was obtained as a concentration (e.g., mg/ml). The values of eight samples (or less if an analyte could not be analyzed in all samples because of insufficient quantity of the sample) were averaged and compared to the sensitivity of the system. An analyte was considered “detectable” if the levels exceeded the minimal detectable levels. The standard deviation for the detected analytes was calculated using an Excel® (Microsoft, Redmond, VA) spreadsheet. The pre-treatment levels were compared with previously published data on normal aqueous samples that were obtained from patients undergoing cataract surgery without any other ocular pathology. The data on normal samples was previously published [5]. Since the microbead assay is highly reproducible [6], it is therefore possible to compare values from different assays. Thus, the previously published results were used as a control for pre- and post-treatment samples. The pre-treatment samples were also compared with the post-treatment samples to elucidate the effect of intravitreal bevacizumab injections. Further, cluster analysis was performed on the analytes detected in all three conditions (normal, pre-treatment, and post-treatment). For cluster analysis, the gene ID for the analytes was identified and log values of the data were used to import them to Cluster and Treeview software. Red indicates upregulated analytes and green indicates low expression. The purpose of cluster analysis was to provide an easily appreciable global view of changes in analyte values in normal, pre-treatment and post-treatment samples.

### Statistical analysis

Tukey’s post-hoc test was used to determine the significant differences in two groups of analytes (pre-treated versus normal and post-treated versus normal). Unsupervised hierarchical average-link clustering using Cluster and Treeview (Eisen Laboratory, Stanford University, Palo Alto, CA) was done to find patterns in analyte expression levels.

## Results

**Figure 1:**
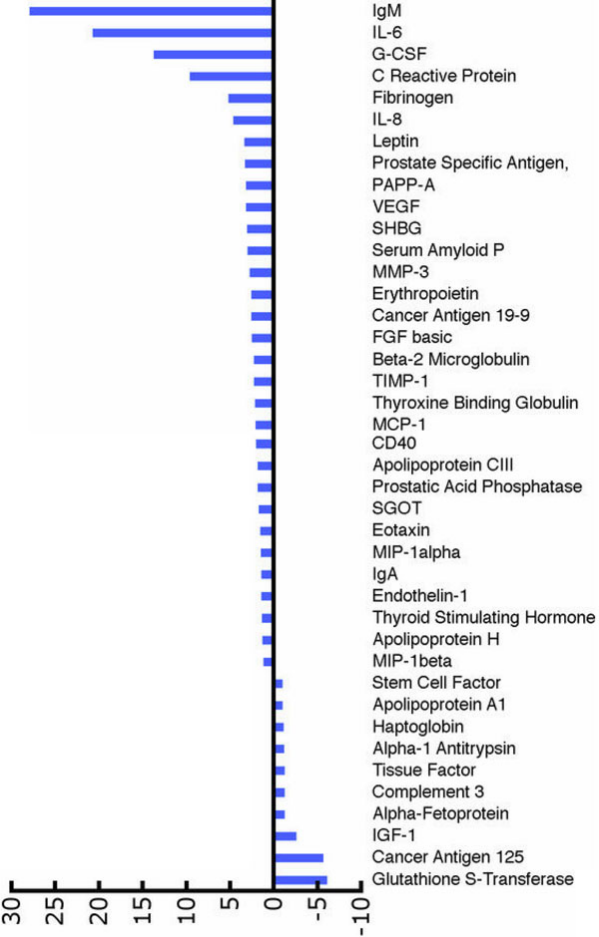
Differences in normal and pathological samples. The figure shows the fold change in the analyte profile in the aqueous humor of patients with VEGF-related pathologies (pre-treatment samples) as compared to known normal values. The 41 analytes that were detected in the aqueous humor of the eyes with VEGF-related pathology were compared with the normal values previously published. The changes are presented as fold change. Ten analytes were down-regulated and 31 were upregulated.

**Figure 2:**
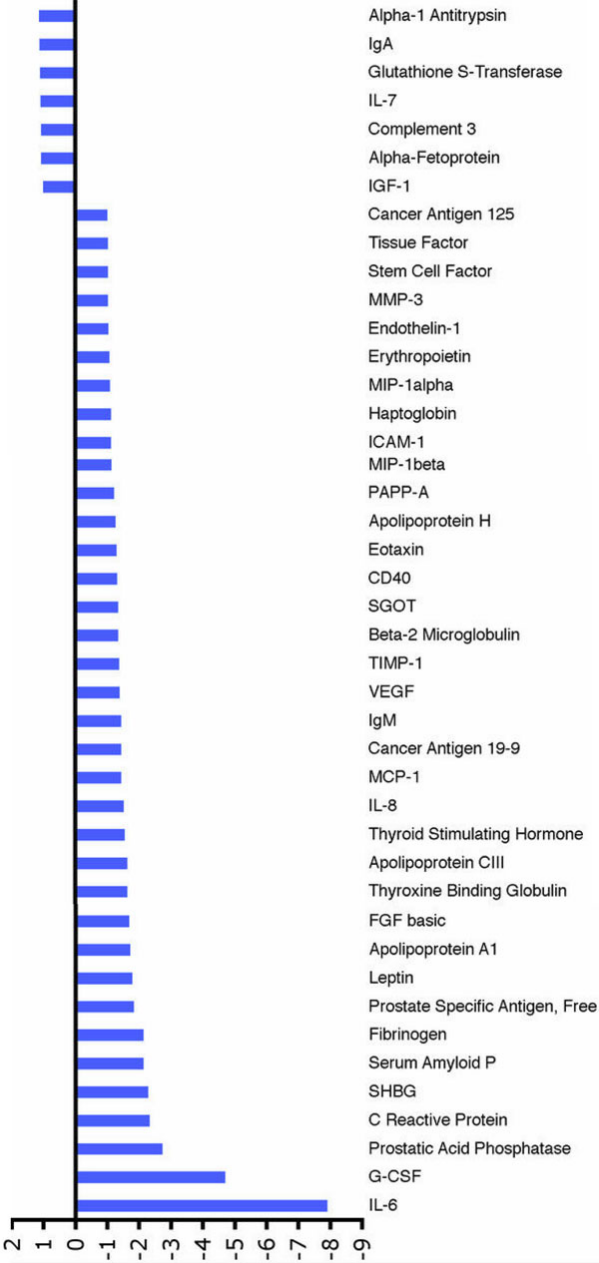
Changes after treatment compared to pretreatment values. The figure shows fold change in the analyte profile in the aqueous humor of patients with VEGF-related pathologies (pre-treatment samples) as compared to the aqueous profile of treated patients (post-treatment). Forty-three analytes were common in pre- and post-treatment samples; out of these, seven analytes were upregulated and the other 36 were down-regulated, suggesting a trend toward normalization.

**Figure 3:**
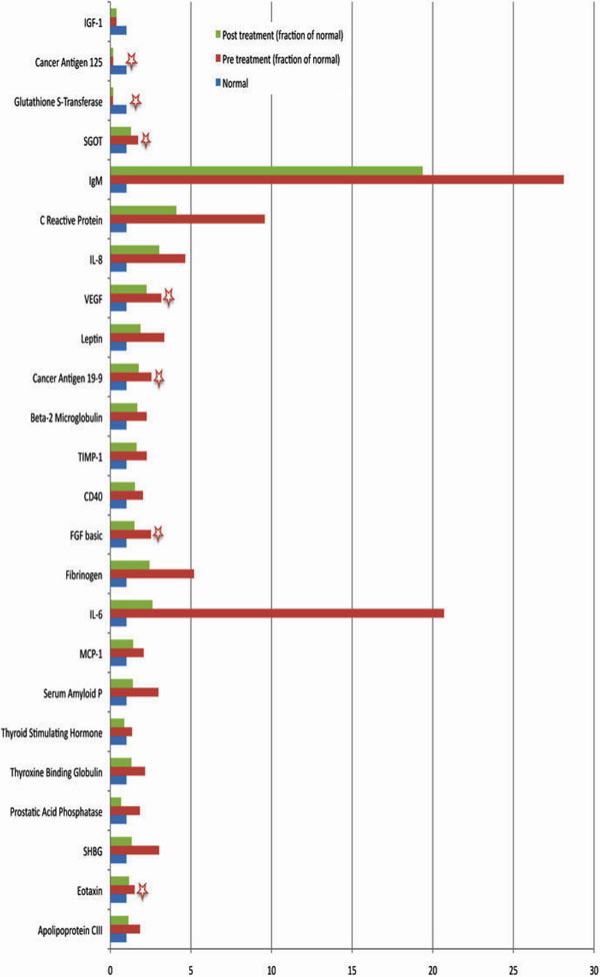
Trend toward normalization of pathological analyte profile after bevacizumab treatment. This figure shows a comparison of 24 analytes that showed a 1.5 fold or more change in the aqueous of eyes with VEGF-related pathology as compared to normal values. The effect of treatment on the analyte profile was followed by analyzing the samples from the same patients after treatment. The figure shows that all analytes upregulated in pre-treatment samples showed a trend toward normalization. Two groups of analytes (pre-treated versus normal and post-treated versus normal) were compared for each analyte using the Tukey’s post-hoc comparison test. Stars show significant differences (p<0.05) between pre-treated versus normal.

A total of 90 analytes represented on MAP analytes were analyzed. Of the 90 analytes, 43 were detected in pre- and post-samples above the detectable limits. Of the 43 analytes, we obtained control values (values from aqueous obtained from non-pathological eyes) for 41. Two analytes (interleukin-7 [IL-7] and inter-cellular adhesion molecule-1 [ICAM-1]) were detected in pre- and post-treatment samples, but not in control samples. A list of analytes detected in all the samples is provided in Table 1. These 41 analytes were further analyzed. Further analysis revealed that of 41 analytes common to the control and pre-treatment samples, 10 were down-regulated in pre-treatment samples and 31 were upregulated (Figure 1). Of the 43 analytes common to pre- and post-treatment samples, seven analytes were upregulated compared to the pre-treatment samples and the other 36 were down-regulated, suggesting a trend toward normalization (Figure 2). To further confirm this observation, the 41 analytes that were common to pre- and post-treatment samples and were also reported in normal samples were individually compared to see if the changes observed in the pre-treatment samples (aqueous from diseased eyes) as compared to control samples (normal values) were reversed after bevacizumab injection in post-treatment samples. Toward this goal, we identified 24 analytes that demonstrated a 1.5 fold or more difference (upregulation or down-regulation) in pre-treatment samples as compared to the normal samples; then we compared this change to the post-treatment samples. This cut-off limit was chosen to eliminate analytes that might have shown some differences merely by chance when compared to controls. Values between a 1.5 and threefold change are generally used in gene expression studies [7-9]. Although these studies relate to microarray analysis, it is reasonable to employ similar cut-off limits to other studies such as this. The upregulated analytes included IL-6, IL-8, erythropoietin, basic fibroblast growth factor (bFGF), CD40, C-reactive protein, fibronigen, VEGF, and tissue inhibitor of metalloproteinase (TIMP-1). The down-regulated analytes included glutathione s-transferase, complement 3, and insulin-like growth factor (IGF-1). In all the 21 upregulated pre-treated analytes, there was a trend toward normalization of the values (Figure 3). All three down-regulated pre-treated analytes did not show any response to treatment.

**Table 1:** List of analytes detected in pathological aqueous samples (pre-treatment) and after intravitreal bevacizumab injection (post-treatment).

**Analyte**	**Units**	**Pre-treatment mean**	**Pre-treatment SD**	**Post-treatment mean**	**Post-treatment SD**
Alpha-1 Antitrypsin	mg/ml	0.0022	0.0012	0.0025	0.0011
Alpha-Fetoprotein	ng/ml	0.2626	0.0569	0.2790	0.0636
Apolipoprotein A1	mg/ml	0.0007	0.0008	0.0004	0.0004
Apolipoprotein CIII	ug/ml	0.0424	0.0188	0.0259	0.0032
Apolipoprotein H	ug/ml	0.8176	0.7303	0.6400	0.5762
Beta-2 Microglobulin	ug/ml	0.4088	0.3180	0.3026	0.1879
C Reactive Protein	ug/ml	0.1114	0.1521	0.0477	0.0514
Cancer Antigen 125	U/ml	1.7363	0.4314	1.7225	0.3476
Cancer Antigen 19–9	U/ml	0.2952	0.1282	0.2042	0.1177
CD40	ng/ml	0.0676	0.0598	0.0510	0.0334
Complement 3	mg/ml	0.0013	0.0007	0.0014	0.0010
Endothelin-1	pg/ml	5.4863	1.7494	5.2375	0.6159
Eotaxin	pg/ml	17.7750	4.6849	13.5813	5.1661
Erythropoietin	pg/ml	120.5875	125.032	111.6125	130.0555
FGF basic	pg/ml	52.2375	18.8596	30.8900	20.8668
Fibrinogen	mg/ml	0.0011	0.0018	0.0005	0.0005
G-CSF	pg/ml	17.2825	31.2826	3.6748	2.8391
Glutathione S-Transferase	ng/ml	0.1406	0.0164	0.1544	0.0154
Haptoglobin	mg/ml	0.0005	0.0005	0.0004	0.0005
IgA	mg/ml	0.0014	0.0011	0.0015	0.0016
IGF-1	ng/ml	1.4248	0.7489	1.4285	0.8083
IgM	mg/ml	0.0013	0.0012	0.0009	0.0008
IL-6	pg/ml	120.2543	293.001	15.2016	19.9646
IL-8	pg/ml	22.9475	30.8635	14.9900	9.5664
Leptin	ng/ml	0.3806	0.5658	0.2131	0.3830
MCP-1	pg/ml	638.0000	618.982	438.3750	263.5244
MIP-1alpha	pg/ml	10.3625	3.0867	9.4175	2.4535
MIP-1beta	pg/ml	24.8738	20.2587	21.7988	14.5772
MMP-3	ng/ml	0.6362	1.2937	0.6159	0.4960
PAPP-A	U/ml	0.0526	0.0558	0.0432	0.0306
Prostate Specific Antigen, Free	ng/ml	0.0169	0.0066	0.0092	
Prostatic Acid Phosphatase	ng/ml	0.0260	0.0273	0.0095	0.0054
Serum Amyloid P	ug/ml	0.0287	0.0303	0.0134	0.0081
SGOT	ug/ml	1.8149	0.6531	1.3488	0.3111
SHBG	nmol/l	0.5133	0.3151	0.2250	0.0866
Stem Cell Factor	pg/ml	19.9938	6.1891	19.4375	2.5757
Thyroid Stimulating Hormone	U/ml	0.0293	0.0252	0.0188	0.0107
Thyroxine Binding Globulin	ug/ml	0.6370	0.6607	0.3876	0.3822
TIMP-1	ng/ml	30.9825	29.6818	22.3825	14.6893
Tissue Factor	ng/ml	0.3724	0.0820	0.3631	0.0698
VEGF	pg/ml	679.6250	299.134	484.1250	346.3955
ICAM-1	ng/ml	0.7900	0.7117	0.6971	0.4189
IL-7	pg/ml	11.4175	1.5109	12.3125	1.1753

This shows the mean values and standard deviation (SD) of 41 analytes that were detectable and for which control values were available. SD=Standard Deviation.

**Figure 4:**
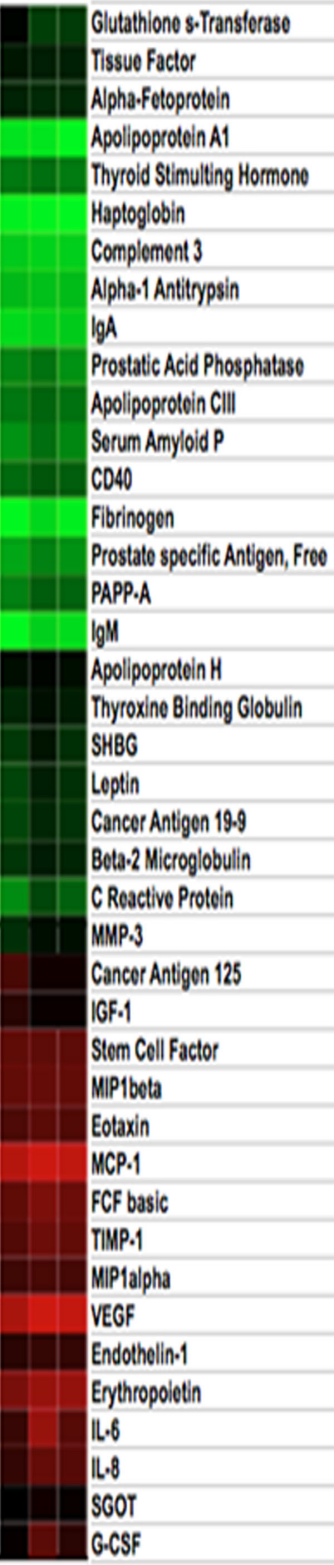
Cluster analysis to provide a global view of changes in all 41 analytes in pre- and post-treatment samples as compared to normal. Log values were used for importing data to Cluster and Treeview software. Red indicates upregulated analytes; green indicates low expressed analytes. The left column shows control values, the middle column shows post-treatment values, and the right column shows pre-treatment values.

Further cluster analysis confirmed that bevacizumab treatment normalized the expression level of detected analytes toward normal values, as published earlier [5] (Figure 4).

## Discussion

Multi-analyte profiles are a fully validated technique [10] based on Luminex technology. This assay performs up to 100 multiplexed, microsphere-based assays in a single reaction vessel by combining optical classification schemes, biochemical assays, flow cytometry, and advanced digital signal processing hardware and software. Research Based Medicine’s MAPs represent the number of markers relevant to neovascular pathology such as hormones, cytokines/chemokines, acute phase reactants, clotting proteins, growth factors, tissue modeling factors, and other typical plasma proteins [11]. Using MAPs to measure a broad spectrum of biomarkers, a comprehensive analysis of intraocular physiologic status can be obtained [5]. We hypothesized that such an analysis could be useful in understanding the pathobiology of a disease, assessing its progression, or assessing the response to a treatment. We provided the proof of principle by comparing two diabetic samples and demonstrating relevant differences in aqueous profiles [5]. In the current study, we used this technique to study how an aqueous profile consisting of 90 analytes represented on the MAP differed in pathological samples compared to previously published values from non-neovascular (normal) samples. We further analyzed post-treatment samples from the same patients to see whether the aqueous humor profile normalized after intravitreal injection. Using the same multiplex in our previous study, we provided baseline values of 52 analytes. Since microbead assays are highly reproducible, it permits inter-experiment comparison [6]. Therefore, we were able to compare pre- and post-treatment values with previously published normal values. Many of the detected analytes such as IL-6, IL-8, granulocyte colony-stimulating factor (G-CSF), fibrinogen, VEGF, matrix metalloproteinase 3 (MMP3), erythropoietin, bFGF, TIMP-1, CD40, C reactive protein, endothelin, and glutathione S-transferase are implicated in ocular pathologies and therefore their measurement could provide useful information [12-15]. The changes observed in pathological samples were consistent with the neovascular and permeability-related pathophysiology. Analytes relevant to neovascular pathology that showed increased levels included IL-6, C reactive protein, fibrinogen, IL-8, VEGF, MMP-3, erythropoietin, CD40, bFGF, and TIMP-1 [16,17]. Equally relevant for neovascular pathophysiology were glutathione S and IGF-1, which were down-regulated in aqueous samples [18,19]. It may be noted that the standard deviations for many analytes in this study were relatively large. This reflects the wide range of analyte values among different patients. We did not make any effort to control the recruitment of patients based on the level of analyte. Therefore, the level of analytes present in the aqueous varies widely between the individuals (range was large), reflecting individual variation, disease process, and the stages of disease.

Most significantly, this study demonstrated that the effect of anti-VEGF therapy on the in oculo pathophysiological environment extended beyond neutralization of VEGF [20]. We identified 24 analytes that were distinctly changed (1.5 fold or more) in the aqueous from the pathological samples as compared to normal values. Analysis of these analytes in the same patients after the bevacizumab injection demonstrated that all analytes upregulated in pre-treatment tended to normalize after the treatment. Many of these analytes are not directly related to the function of VEGF. These results provide strong and unequivocal evidence that the bevacizumab treatment affects the pathophysiological state of the eye beyond what could be expected by neutralization of VEGF alone. These results were confirmed by a global view of the changes provided by the cluster analysis. The purpose of cluster analysis in this study was not to group analytes, but rather to use the color coding that expresses the analyte levels to show how all the analytes in Table 1 changed in three different conditions. It is apparent from looking at Figure 4 that bevacizumab treatment had a wide-ranging impact on the aqueous profile toward normalization. It is possible that some of the changes noted may be the result of the regression of the disease process (indirect effect of the treatment) or the reestablishment of the blood-retinal barrier rather than the direct effect of the bevacizumab injection.

Clinical observations suggest that the bevacizumab treatment changes the clinical course of neovascularization in a manner that cannot be entirely explained by the VEGF neutralizing effect of bevacizumab. For instance, intracameral injection of bevacizumab for iris neovascularization lasts longer than bevacizumab availability in the aqueous [20] considering aqueous is produced at a rate of 2–3 µl/min and the volume of anterior chamber is approximately 250 µl. Similarly, the effect of intravitreal injection of bevacizumab lasts for months, longer than the bioavailability of bevacizumab [21,22]. In addition, bevacizumab treatment not only arrests the progression of neovascularization, but in many cases reverses it, which again cannot be explained by the effect of VEGF on endothelial cell proliferation and migration alone [20]. Our results provide clues that the trend toward the global normalization of the ocular physiologic environment by bevacizumab injection could alter the pathophysiology of the disease process and thus potentially provide an explanation for these observations.

One of the limitations of the study was that the MAPs we used were not specifically designed for ocular pathologies. However, based on this technology, ocular pathology-specific MAPs could be designed. These results suggest that expression profiling of the aqueous by specifically designed MAPs could provide valuable insight into the disease processes and treatment responses using a systems approach. Such an approach could facilitate deduction of the complex networks that underlie biologic processes and characterization of their state when perturbed by disease [23]. This approach would aid understanding of the various aspects of diseases, including their classification, pathobiology, treatment responses, and prognoses [24] as well as open the door for personalized, tailored strategies for ocular diseases at all levels of management.
